# Association of elevated α-defensin levels with interstitial pneumonia in patients with systemic sclerosis

**DOI:** 10.1186/s12931-015-0308-1

**Published:** 2015-12-10

**Authors:** Noriho Sakamoto, Tomoyuki Kakugawa, Atsuko Hara, Shota Nakashima, Hirokazu Yura, Tatsuhiko Harada, Hiroshi Ishimoto, Kazuhiro Yatera, Yutaka Kuwatsuka, Toshihide Hara, Kunihiro Ichinose, Yasushi Obase, Yuji Ishimatsu, Shigeru Kohno, Hiroshi Mukae

**Affiliations:** Second Department of Internal Medicine, Nagasaki University School of Medicine, 1-7-1 Sakamoto, Nagasaki, 852-8501 Japan; Department of Respiratory Medicine, University of Occupational and Environmental Health, Kitakyushu, Japan; Department of Dermatology, Graduate School of Medicine, Nagasaki University, Nagasaki, Japan; Department of Immunology and Rheumatology, Nagasaki University Graduate School of Biomedical Sciences, Nagasaki, Japan; Department of Cardiopulmonary Rehabilitation Science, Unit of Rehabilitation Sciences, Nagasaki University Graduate School of Biomedical Sciences, Nagasaki, Japan

**Keywords:** Bronchoalveolar lavage fluid, Human neutrophil peptide, Interleukin-8, Interstitial lung disease

## Abstract

**Background:**

Interstitial lung disease (ILD) is the leading cause of mortality in patients with systemic sclerosis (SSc). Although the pathogenesis of SSc-ILD is not well understood, neutrophils may play a pivotal role in this process. Neutrophils store azurophil granules that contain defensins, antimicrobial peptides that function in regulating the inflammatory response, and IL-8, a potent chemoattractant for neutrophils. The present study evaluated the levels of defensins and IL-8 in patients with SSc-ILD to determine their roles in disease pathogenesis.

**Methods:**

Defensins (also known as human neutrophil peptides, HNPs) and IL-8 levels were measured in the serum and bronchoalveolar lavage fluid (BALF) of 33 patients with SSc-ILD and in 20 healthy controls by using ELISA.

**Results:**

BALF analysis revealed a significant increase in HNPs in SSc-ILD patients (median; 240.0 pg/mL) than that of healthy controls (79.7 pg/mL). Additionally, IL-8 levels were higher in SSc-ILD patient serum and BALF as compared to healthy controls (16.4 pg/mL vs. 5.8 pg/mL and 15.4 pg/mL vs. 14.5 pg/mL, respectively). However, plasma HNPs levels were relatively unchanged. HNP and IL-8 levels in patient BALF displayed a significant positive correlation significantly correlated (r = 0.774, *p* <0.01), and which also correlated with clinical disease parameters—such as ILD biomarkers, pulmonary function tests, ratio of neutrophils and eosinophils in BALF, tricuspid regurgitation peak gradient (TRPG), and the extent of high-resolution computed tomography (HRCT) findings in the lung. Levels of plasma HNPs and serum IL-8 did not show a significant correlation with any clinical parameter. SSc-ILD progression was evaluated by pulmonary function tests, but no association was observed between VC change ratios and HNPs or IL-8 levels.

**Conclusions:**

BALF levels of HNPs and IL-8 were higher in SSc-ILD than in healthy controls, and are associated with various clinical disease parameters. Further studies are needed to clarify the role of defensins and IL-8 in SSc-ILD pathogenesis.

## Background

Systemic sclerosis (SSc) is a heterogeneous disease characterized by small vessel vasculopathy, autoantibody production, and fibroblast dysfunction leading to increased extracellular matrix deposition [1]. SSc often affects multiple systems, including the skin and visceral organs. Pulmonary involvement is prominent in SSc, and interstitial lung disease (ILD) is the leading cause of mortality in SSc patients [2]. Although SSc-ILD pathogenesis is not well understood, the aberrant function of a variety of lung cells, cytokines, growth factors, peptides, and bioactive proteins, in combination with genetic and epigenetic regulators, have crucial functions in this process [3]. High-resolution computed tomography (HRCT) is the standard method for the noninvasive diagnosis of SSc-ILD and can detect mild abnormalities [4]. The extent of disease observed with HRCT and lower diffusing capacity of carbon monoxide (DLco) are reported to be associated with poor SSc-ILD prognosis [5, 6]. Although the cellular analysis of bronchoalveolar lavage fluid (BALF) is limited to excluding infection [4], it has been reported that neutrophilia in BALF is associated with disease severity [7].

IL-8 is a potent neutrophil chemoattractant. As such, elevated IL-8 levels in BALF often correlate with pulmonary neutrophil accumulation and poor function tests in SSc-ILD patients [8], as well as extensive fibrosis in HRCT [9]. Correspondingly, IL-8 mRNA is shown to be upregulated in fibroblasts derived from localized scleroderma lesions in these patients [10, 11]. Thus, these data support a possible role of IL-8 in SSc-ILD pathogenesis.

Defensins are small, arginine-rich cationic peptides with antimicrobial activity [12]. Humans express α- and β-defensins. Among the six known α-defensins, human neutrophil peptides (HNP)-1 to 4 are mainly found in neutrophils, whereas human defensins (HD)-5 and HD-6 are primarily expressed in intestinal Paneth cells, and the respiratory and female reproductive tracts [13]. Human β defensins (HBDs) are expressed by epithelial cells in the skin and at mucosal surfaces in contact with the environment. Defensins play antimicrobial roles, and might also function in regulating inflammatory responses [14]. A series of studies have found increased HNPs and HBD levels in plasma or BALF from patients with various inflammatory lung diseases [15, 16]. Moreover, we previously reported that elevated HNP levels in BALF were associated with heighted neutrophil counts and IL-8 in patients with several lung diseases [17–21].

Here, we evaluated the concentrations of HNPs and IL-8 in the BALF and blood of patients with SSc-ILD to determine their roles in disease pathogenesis.

## Methods

### Study population

The study population consisted of 33 SSc-ILD patients at Nagasaki University Hospital between 2000 and 2014 and 20 healthy volunteers. All SSc patients fulfilled the American College of Rheumatology classification criteria [22]. SSc-ILD was diagnosed by HRCT of the lung, and one of these patients was pathologically diagnosed as fibrotic nonspecific interstitial pneumonia (NSIP) by surgical lung biopsy. BALF and blood samples were collected at their first visit and stored at −20 °C until use. No patient was treated by systemic steroid and/or immunosuppressants at the sample collection. All data, including those from pulmonary function tests, arterial blood gas analyses, markers of interstitial pneumonia—such as Krebs von den Lungen 6 (KL-6), surfactant protein (SP)-A, and SP-D, and survival rates were obtained from medical records. Screening for pulmonary hypertension was performed by transthoracic echocardiography in 30 of 33 patients, and tricuspid regurgitation peak gradient (TRPG) was used as indicator of pulmonary hypertension. All healthy controls were asymptomatic, not taking any medication, and had normal chest radiographs. All participants provided written, informed consent before enrollment. The study protocol was approved by the Human Ethics Review Committee at Nagasaki University School of Medicine.

### Evaluation of ILD

All HRCTs were obtained at the time of diagnosis and assessed retrospectively. The extent of visual ground glass opacity, reticular opacities, and honeycombing were determined by visually estimating the extent in the upper, middle, and lower zones of each lung based on the percentage of the lung field that showed each abnormality in each zone (estimated to the nearest 10 % of parenchymal involvement) according to the reports of Johkoh et al. [23] and Sumikawa et al. [24]. The upper zone was defined as the area above the level of the carina; the lower zone as the area below the level of the inferior pulmonary vein; and the middle zone as the area between the upper and lower zones. Overall percentage of involvement was obtained by averaging the six lung zones. BALF was collected with three instillations of sterile physiological saline (50 mL) through a flexible bronchoscope as previously described [25]. The collected lavage fluid was passed through two sheets of gauze and centrifuged at 400 × *g* for 10 min at 4 °C, and the supernatant stored at −20 °C until use. ILD progression was evaluated by the percentage of relative decline in absolute VC [26].

### Measurement of HNPs and IL-8

HNP and IL-8 concentrations in serum and BALF were measured using sandwich enzyme-linked immunosorbent assay (ELISA) kits according to the manufacturers’ protocols (HNP1-3, HyCult Biotechnology, Uden, Netherlands; R&D Systems, Minneapolis, MN, USA). Samples were diluted 1000-fold for plasma HNPs, and 5-fold for serum and BALF IL-8. The lower limit of detection was 156 pg/mL for HNPs and 3.5 pg/mL for IL-8. Assays were performed in duplicate, and inter-/intra-assay coefficients of variation for plasma HNPs, BALF HNPs, serum IL-8, and BALF IL-8 were 10.6/6.3, 10.7/10.6, 12.4/7.9 and 7.8/11.9 %, respectively.

### Statistical analysis

All values are expressed as the median and inter-quartile range (IQR). Differences between and among groups were compared using the Mann-Whitney U and Kruskal-Wallis tests, respectively. Correlations between parameters were determined by Spearman’s rank correlation coefficient. *P* <0.05 was regarded as statistically significant.

## Results

### Patients characteristics

Patient demographic and clinical data are shown in Table 1. The median patient age was 63, and 10 of 33 patients were men. Several patients had coexisting rheumatic diseases. Laboratory findings in patients with SSc-ILD are shown in Table 2.

**Table 1:** Characteristics of patients with SSc-ILD

	*N* = 33	
Gender (Male/Female)	10/23	
Age (Years)	63	(54–70)
Duration from onset (Months)	36	(10–84)
Smoking (Non/Ex/Current)	22/8/3	
Auto-antibodies		
Anti-topoisomerase I Ab	17	(52 %)
Anti-centromere Ab	6	(18 %)
Anti-RNA polymerase Ab	2	(6 %)
Anti-Th/To Ab	1	(3 %)
Anti-RNP Ab	1	(3 %)
Unknown	6	(18 %)
Coexisting rheumatic disease		
Sjögren's syndrome	4	(12 %)
Primary biliary cirrhosis	2	(6 %)
Sarcoidosis	2	(6 %)
Rheumatoid arthritis	1	(3 %)
Systemic lupus erythematosus	1	(3 %)
Polymyositis	1	(3 %)

**Table 2:** Laboratory findings in patients with SSc-ILD

Variables	*N*	median	IQR
Laboratory data			
PaO2 (torr)	28	92.0	(76.6–95.1)
KL-6 (U/mL)	30	973	(529–1986)
SP-D (ng/mL)	33	212	(114–308)
SP-A (ng/mL)	25	83.4	(58.3–121.5)
Pulmonary function test			
%VC (%)	33	92.3	(76.4–111.8)
FEV1/FEV (%)	33	78.4	(73.5–83.7)
%DLco (%)	32	56.6	(41.2–66.9)
BALF cell findings			
TCC (× 10^5^/mL)	33	2.8	(2.3–4.4)
Macrophages (%)	33	74.8	(58.3–86.8)
Lymphocytes (%)	33	10.6	(7.9–18.7)
Neutrophils (%)	33	5.1	(1.8–12.0)
Eosinophils (%)	33	1.7	(1.0–5.9)
CD4/8 (%)	33	1.3	(0.6–2.2)
HRCT findings			
Ground glass opacity (%)	33	11.7	(5.9–29.2)
Reticular opacities (%)	33	6.7	(1.7–15.9)
TRPG	30	25.5	(18.0–30.5)

*IQR* inter-quartile range, *TRPG* tricuspid regurgitation peak gradient

### Plasma, serum, and BALF levels of HNPs and IL-8

**Fig. 1:**
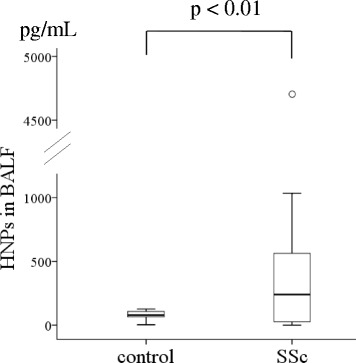
Human neutrophil peptide (HNP) levels in the BALF of SSc-ILD patients. HNP levels were analyzed in BALF of patients with systemic sclerosis associated interstitial lung disease (SSc-ILD) and healthy controls by ELISA. The data is represented by Tukey boxplots

**Fig. 2:**
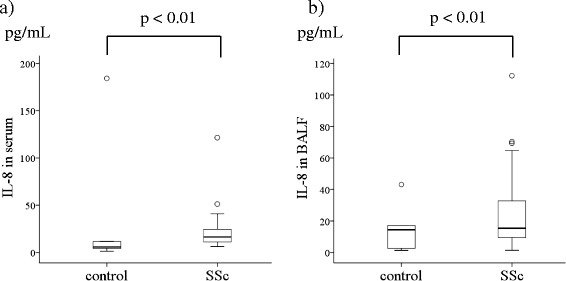
IL-8 levels in SSc-ILD patient serum and BALF. IL-8 levels were analyzed in the serum (**a**) and BALF (**b**) of SSc-ILD patients and healthy controls by ELISA. The data is represented by Tukey boxplots

HNP analysis revealed significantly higher levels in the BALF of SSc-ILD patients (median, 240.0 pg/mL; IQR, 26.4–563.1 pg/mL) than that of healthy controls (79.7 pg/mL, 65.6–107.8 pg/mL, *p* <0.01, Fig. 1); however, no differences were observed in plasma HNPs levels (65.9 ng/mL, 27.0–79.9 ng/mL) and SSc-ILD (101.0 ng/mL, 58.35–141.3 ng/mL, *p* = 0.20). Alternatively, IL-8 levels were higher in SSc-ILD patient serum (16.4 pg/mL, 11.3–24.6 pg/mL vs. 5.8 pg/mL, 4.4–11.7 pg/mL in healthy controls, *p* <0.01; Fig. 2a) and BALF (15.4 pg/mL, 9.5–32.8 pg/mL vs. 14.5 pg/mL, 2.7–17.1 pg/mL in healthy controls, *p* <0.01; Fig. 2b).

### Association between HNPs, IL-8, and clinical parameters in SSc-ILD

**Fig. 3:**
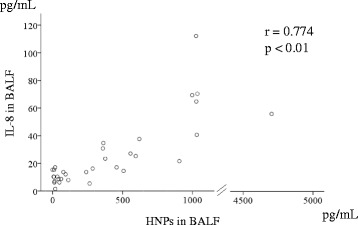
Correlation between IL-8 and HNP levels in BALF samples from SSc-ILD patients

**Fig. 4:**
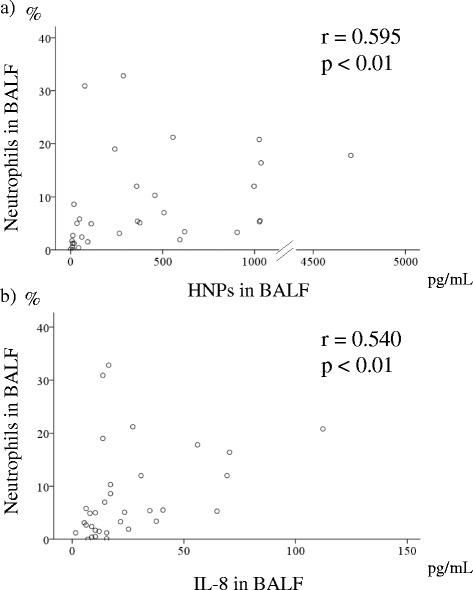
Correlation between (HNPs; **a**) or IL-8 (**b**) levels and neutrophil percentage in BALF samples from SSc-ILD patients

**Fig. 5:**
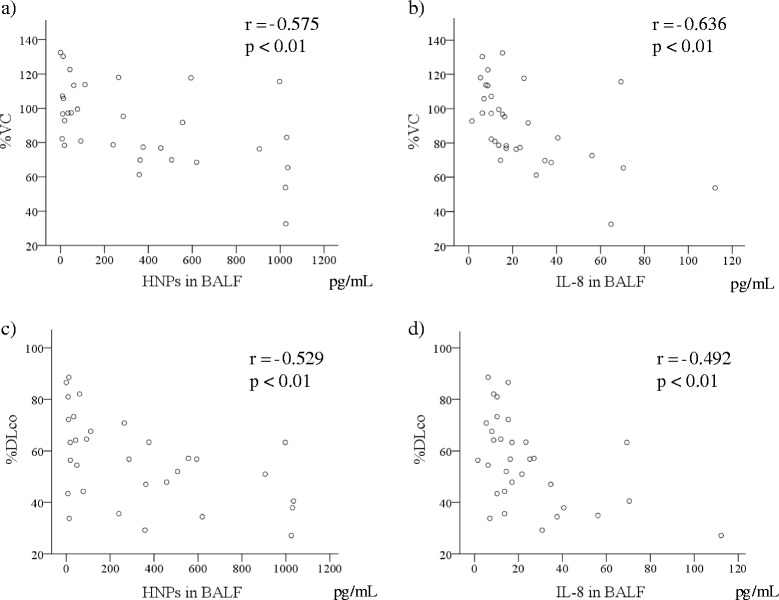
Correlation between (HNPs; **a, c**) or IL-8 (**b, d**) BALF levels and pulmonary function tests in SSc-ILD patients

We then analyzed the relationships between clinical parameters and HNPs and IL-8 levels in the serum and BALF of SSc-ILD patients (Table 3). Notably, the levels of BALF HNPs and BALF IL-8 correlated significantly (r = 0.774, Fig. 3). Both BALF HNPs and IL-8 levels were positively correlated with serum KL-6 (HNPs; r = 0.511, IL-8; r = 0.467), SP-D (r = 0.363, r = 0.360), and the percentage of neutrophils (r = 0.595, r = 0.540; Fig. 4) and eosinophils (r = 0.466, r = 0.419) in BALF, and negatively correlated with PaO_2_ (r = −0.416, r = −0.429), VC (r = −0.575, r = −0.636), and DLco (r = −0.529, r = −0.492; Fig. 5). Moreover, both HNPs and IL-8 BALF levels were positively correlated with TRPG (r = 0.517, r = 0.473). Levels of plasma HNPs and serum IL-8 did not show a significant correlation with any clinical parameters. SSc-ILD progression was evaluated by the percentage of relative decline in absolute VC in 15 patients over 1 year and 13 of 32 patients for 2 years, but no associations were observed.

**Table 3:** Correlation between HNPs and IL-8 and clinical parameters in patients with SSc-ILD

Variables		HNPs in BALF			IL-8 in BALF		
	*N*	r	95 % CI	*P*-value	r	95 % CI	*P*-value
HNPs in plasma	33	0.049	−0.299 – 0.386	0.79	−0.06	−0.395 – 0.289	0.75
HNPs in BALF	33	–	–	–	0.774	0.587 – 0.883	<0.01
IL-8 in serum	33	0.324	−0.022 – 0.600	0.07	0.160	−0.194 – 0.477	0.38
IL-8 in BALF	33	0.774	0.587 – 0.883	<0.01	–	–	–
Laboratory data							
PaO2 (torr)	28	−0.416	−0.683 – −0.051	0.03	−0.429	−0.691 – −0.067	0.02
KL-6 (U/mL)	30	0.511	0.185 – 0.736	<0.01	0.467	0.128 – 0.708	<0.01
SP-D (ng/mL)	33	0.363	0.023 – 0.628	0.04	0.360	0.019 – 0.626	0.04
SP-A (ng/mL)	25	0.256	−0.155 – 0.591	0.23	0.364	−0.36 – 0.664	0.08
Pulmonary function test							
%VC (%)	33	−0.575	−0.767 – −0.289	<0.01	−0.636	−0.804 – −0.374	<0.01
FEV1/FEV (%)	33	0.106	−0.246 – 0.434	0.56	−0.091	−0.421 – 0.260	0.61
%DLco (%)	32	−0.529	−0.741 – −0.221	<0.01	−0.492	−0.718 – −0.173	<0.01
BALF cell findings							
TCC (× 10^5^/mL)	33	−0.036	−0.375 – 0.311	0.84	−0.065	−0.399 – 0.285	0.72
Macrophages (%)	33	0.016	−0.329 – 0.357	0.93	−0.010	−0.352 – 0.335	0.96
Lymphocytes (%)	33	−0.153	−0.472 – 0.201	0.40	−0.167	−0.483 – 0.187	0.35
Neutrophils (%)	33	0.595	0.316 – 0.779	<0.01	0.540	0.242 – 0.745	<0.01
Eosinophils (%)	33	0.466	0.146 – 0.698	<0.01	0.419	0.088 – 0.666	0.02
CD4/8 (%)	33	−0.090	−0.420 – 0.261	0.62	−0.009	−0.351 – 0.335	0.96
HRCT findings							
Ground glass opacity (%)	33	0.711	0.486 – 0.848	<0.01	0.583	0.230 – 0.772	<0.01
Reticular opacities (%)	33	0.652	0.398 – 0.813	<0.01	0.605	0.330 – 0.785	<0.01
TRPG	30	0.517	0.193 – 0.740	<0.01	0.473	0.136 – 0.712	<0.01
Decline in absolute VC (%)							
One year	15	−0.275	−0.690 – 0.276	0.32	−0.059	−0.555 – 0.467	0.84
Two years	12	−0.070	−0.619 – 0.525	0.83	0.010	−0.567 – 0.581	0.97

*TRPG* tricuspid regurgitation peak gradient

## Discussion

In the present study, we found elevated levels of BALF HNPs, BALF IL-8, and serum IL-8 in SSc-ILD patients when compared with healthy controls, but no significant differences were observed with respect to plasma HNPs. Both BALF HNPs and IL-8 levels were associated with clinical parameters, such as markers of interstitial pneumonia (KL-6, SP-D), pulmonary function tests, ratios of neutrophils and eosinophils in BALF, and the extent of lung HRCT findings. Notably, this is the first study to suggest that HNPs in BALF play a primary role in SSc-ILD pathophysiology.

SSc-ILD severity is reported to be associated with BALF IL-8 levels [8]. This was supported by our data, which also added a correlation with BALF HNPs. Moreover, both HNPs and IL-8 in BALF correlated with neutrophil number in the present study. It has been reported that SSc-ILD severity associated with neutrophilia in BALF [5]. BALF HNPs and IL-8 levels also showed a positive correlation. We previously reported that HNPs and IL-8 levels in BALF showed a positive correlation in patients with idiopathic pulmonary fibrosis (IPF) [18]. Furthermore, HNPs induce the production of cytokines—such as IL-8—and growth factors, which play pivotal roles in pulmonary fibrosis, in lung fibroblasts and epithelial cells, as well as the production of collagen by lung fibroblasts in vitro [12, 27, 28]. Altogether, these results suggest that HNPs produced by neutrophils induce IL-8 production in the lung and play a pivotal role in SSc-ILD.

The association between neutrophilia in BALF and disease progression in SSc-ILD is controversial. Crestani et al. reported that spontaneous IL-8 secretion by alveolar macrophages was higher in patients with SSc than control subjects, and positively correlated with the percentage of neutrophils in BALF [29]. From this, they concluded that alveolar macrophages contribute to the influx of neutrophils in alveoli through the release of IL-8. Moreover, Goh et al. reported that neutrophilia in BALF is linked to early mortality, but not to long-term survival or the progression of lung disease [7]. Alternatively, De Santis et al. reported that the neutrophilia in BALF is significantly associated with overall mortality, but is not an independent risk factor of mortality [30]. Our data did not show any association between disease progression and HNPs, IL-8, or neutrophils in the BALF of SSc-ILD patients. Nevertheless, our present results and others [5, 30] indicate that not only neutrophils, but also HNPs and IL-8 in BALF associate with SSc-ILD severity, but not disease progression. Thus, further studies are needed to clarify the association among HNPs, IL-8 and neutrophils in SSc-ILD.

In contrast to BALF HNPs, plasma HNPs levels were not elevated in SSc-ILD nor did they show any correlations with clinical parameters. Our previous reports found elevated plasma HNPs in patients with bacterial infection, acute respiratory distress syndrome, IPF, diffuse panbronchiolitis, and pulmonary tuberculosis [18–20, 31, 32], but not with pulmonary non-tuberculous Mycobacterial infections [21]. Plasma HNPs were also negatively correlated with pulmonary function tests in patients with IPF [18]. Although the precise mechanism of plasma HNP production is not well understood, they are mainly derived from neutrophil precursor cells in bone marrow in response to stimulation by inflammatory mediators [20, 33]. Accordingly, further studies are needed to clarify the differences of plasma HNPs levels between SSc-ILD and other diseases.

Serum IL-8 levels are reported to be elevated and significantly correlated with decreased DLco in SSc patients [34, 35], but did not have the prognostic value with SSc-ILD [36]. In contrast, elevated serum IL-8 levels did not correlate with clinical parameters in SSc-ILD in our study. IL-8 mRNA levels are reported to be upregulated in fibroblasts derived from scleroderma skin lesions [10, 11], suggesting that serum IL-8 may partially mediate SSc-ILD pathology. However, further studies are needed to clarify the role of serum IL-8 levels in disease pathogenesis.

This study has several limitations. We evaluated follow-up data of pulmonary function tests for only 1–2 years with this retrospective study, which could result in an insufficient analysis on the participation of these markers in SSc-ILD progression. Moreover, since the patients were all seen in by the respiratory department, our study only examined IL-8 and HNPs levels in SSc-ILD patients, but not in patients without the ILD component. Thus, we were unable to confirm that lung pathology is responsible for changed levels of IL-8 and HNPs in SSc patients. The small patient cohort is other limitation of this study; thus, a larger patient population and prospective studies are needed for future investigations.

## Conclusions

HNPs in BALF, and IL-8 in serum and BALF were higher in patients with SSc-ILD than healthy controls. While both HNPs and IL-8 in BALF were associated with clinical parameters, further studies are necessary to clarify the role of defensins in SSc-ILD pathogenesis.
